# Hearing in Cichlid Fishes under Noise Conditions

**DOI:** 10.1371/journal.pone.0057588

**Published:** 2013-02-28

**Authors:** Friedrich Ladich, Tanja Schulz-Mirbach

**Affiliations:** Department of Behavioural Biology, University of Vienna, Vienna, Austria; Universität Bielefeld, Germany

## Abstract

**Background:**

Hearing thresholds of fishes are typically acquired under laboratory conditions. This does not reflect the situation in natural habitats, where ambient noise may mask their hearing sensitivities. In the current study we investigate hearing in terms of sound pressure (SPL) and particle acceleration levels (PAL) of two cichlid species within the naturally occurring range of noise levels. This enabled us to determine whether species with and without hearing specializations are differently affected by noise.

**Methodology/Principal Findings:**

We investigated auditory sensitivities in the orange chromide *Etroplus maculatus*, which possesses anterior swim bladder extensions, and the slender lionhead cichlid *Steatocranus tinanti*, in which the swim bladder is much smaller and lacks extensions. *E. maculatus* was tested between 0.2 and 3****kHz and *S. tinanti* between 0.1 and 0.5 kHz using the auditory evoked potential (AEP) recording technique. In both species, SPL and PAL audiograms were determined in the presence of quiet laboratory conditions (baseline) and continuous white noise of 110 and 130 dB RMS. Baseline thresholds showed greatest hearing sensitivity around 0.5 kHz (SPL) and 0.2 kHz (PAL) in *E. maculatus* and 0.2 kHz in *S. tinanti*. White noise of 110 dB elevated the thresholds by 0–11 dB (SPL) and 7–11 dB (PAL) in *E. maculatus* and by 1–2 dB (SPL) and by 1–4 dB (PAL) in *S. tinanti*. White noise of 130 dB elevated hearing thresholds by 13–29 dB (SPL) and 26–32 dB (PAL) in *E. maculatus* and 6–16 dB (SPL) and 6–19 dB (PAL) in *S. tinanti*.

**Conclusions:**

Our data showed for the first time for SPL and PAL thresholds that the specialized species was masked by different noise regimes at almost all frequencies, whereas the non-specialized species was much less affected. This indicates that noise can limit sound detection and acoustic orientation differently within a single fish family.

## Introduction

The auditory sensitivity of fishes has been measured in more than 150 species covering a large number of families, hearing sensitivities and habitats. Fay (1988) [1] reviews 48 species in which baseline hearing abilities have been determined using behavioral techniques, and Ladich and Fay (2012) [2] list 110 species out of 51 families which have been measured using auditory evoked potential (AEP) recording techniques. Almost all species have been measured only under quiet laboratory conditions (although lab noise has not been defined in most studies), making it difficult to assess their ability to detect sound in their habitats. The natural environment of marine and freshwater fishes is characterized by a permanent background noise of abiotic (currents, rain, wind, tides, coastal surf), biotic (vocalizations of animals, underwater movements of plants such as reeds) and increasingly anthropogenic origin (ships and boats, hydrodynamic power plants, seismic exploration). These factors result in a large diversity of ambient noise levels and spectra, which have been characterized recently in a few studies [3]–[12].

The detection of signals is impaired in the presence of other signals such as noise of a certain level – a phenomenon termed masking. Elevated auditory thresholds due to masking have been demonstrated in several fish species and in the presence of various noise types using different techniques (behavioral, AEP). This has been shown using white noise as a masker in otophysines, gadids, batrachoidids, holocentrids, haemulids and centrarchids [13]–[18]. Chapman (1973) [19] and [20] demonstrated in the field in various marine gadiforms (cods) that masking can occur under relatively quiet sea conditions. In otophysines, batrachoidids, gobiids, sciaenids and pomacentrids [21]–[25] masking by field ambient noise proved to be small under quiet conditions (no running water, no wind).

Wysocki and Ladich (2005a) [18] and [21] investigated masking effects in species which differ depending on presence or absence of accessory hearing structures in their auditory sensitivities. They used either white noise as a masker at naturally occurring noise levels or ambient noise recorded in the field (lake, backwater, stream, river) and reported that the decrease in auditory sensitivity (masking effects) was more pronounced in otophysines belonging to cyprinids and doradids (thorny catfishes) than in non-specialized perciforms belonging to centrarchids (sunfishes) and percids (perches). To date, masking phenomena were studied solely in terms of sound pressure; our investigation is the first to also consider particle acceleration.

Previous studies chose hearing specialized and non-specialized species from non-related taxa such as otophysines and perciforms for comparative purposes to study masking. Investigating the effect of different maskers on different hearing sensitivities calls for choosing closely related species, ideally belonging to the same family, that differ considerably in their auditory sensitivities. An intra-familial masking study was carried out only within sciaenids (drums and croakers); it compared two species which, however, did not differ in absolute hearing sensitivities except in the maximum frequency detectable [17]. Pronounced differences in swim bladder morphology and auditory sensitivities have been described only within two (non-related) teleost families, namely holocentrids (squirrelfishes) and cichlids [26]–[29]. In both families some representatives possess anterior extensions of the swim bladder contacting the inner ear, whereas others lack any extensions or even have reduced swim bladders. These differences in morphology result in sensitivity differences of up to 40 dB in both families. Among cichlids, the slender lionhead cichlid *Steatocranus tinanti* (subfamily Pseudocrenilabrinae) has a reduced swim bladder without any connection to the inner ear. In contrast, the orange chromide *Etroplus maculatus* (subfamily Etroplinae) possesses anterior swim bladder extensions touching the cranium at the inner ears [29].

The present study was designed to investigate masking by noise in *S. tinanti* and *E. maculatus*, with special focus on characterizing the hearing thresholds in terms of sound pressure (SPL) as well as particle acceleration levels (PAL). We chose white noise at two different levels within the naturally occurring range as maskers (see [18]) to analyze differences in masking effects among cichlids.

## Materials and Methods

### Study Animals

We measured auditory sensitivities in six specimens of *E. maculatus* (SL, 45±0.8 mm; BW, 3.3±0.19 g) and in four specimens of *S. tinanti* (SL, 52±1.3 mm; BW, 2.4±0.28 g). Fishes originated from local fish suppliers and were transferred to the University of Vienna in August/September 2011 and January 2012. Animals were kept in 98- and 245-l aquaria, which were equipped with a sand bottom, halved flower pots and (artificial) plants as hiding places. Water was maintained using external filters. Fishes were kept under a 12∶12 h L:D cycle at 25±1°C and were fed once daily with commercial flake food and red blood worms.

All hearing experiments were performed with the permission of the Austrian Federal Ministry of Science and Research (permit number GZ 66.006/0023-II/10b/2008).

### Auditory Sensitivity Measurements

Auditory thresholds were determined by applying the AEP recording technique [18], [30]–[32].

In order to reduce muscle noise, the test subjects were immobilized with Flaxedil (gallamine triethiodide; Sigma Aldrich Handels GmbH, Vienna, Austria) at mean concentrations of 4.0 or 14.9 µg*g^–1^ body weight for *S. tinanti* and *E. maculatus*, respectively. All auditory measurements were carried out in an bowl-shaped plastic tub (diameter 37 cm, water depth 16 cm, 0.5 cm layer of sand), which was lined inside with acoustically absorbent material (air-filled packing wrap) to minimize distortions of stimuli (for the effect see figure 1 in [33]). Water temperature and room temperature were kept constant at 25±1°C. *E. maculatus* were tested at 0.2, 0.5, 1, 2 and 3 kHz, *S. tinanti* at 0.1, 0.2, 0.3 and 0.5 kHz. Each specimen was consecutively measured at the above-mentioned frequencies under quiet lab noise conditions, and in the presence of white noise of 110 dB and 130 dB re 1 µPa.

**Figure 1:**
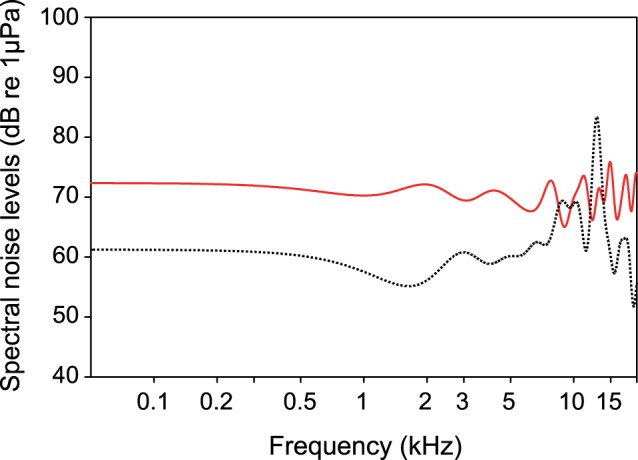
Comparison of cepstrum-smoothed spectra of white noise of 110 dB RMS recorded underwater when using an 30-band equalizer (solid line) and without using an equalizer (dotted line).

### Particle acceleration measurements

In addition to SPL, we determined PAL at thresholds because *S. tinanti* lacks hearing specializations (in contrast to *E. maculatus*) and we therefore assumed that it is mainly sensitive to particle acceleration [32], [34].

In order to determine SPLs and PALs for frequencies up to 1 kHz, a calibrated underwater miniature acoustic pressure-acceleration (p-a) sensor (S/N 2007-001, Applied Physical Sciences Corp., Groton, CT, USA; frequency bandwidth: 20 Hz to 2 kHz; sensitivity: −137.6 dB re 1 V/µm/s^2^ and −173.7 dB re 1 V/µPa) was placed instead of the fish at the position of the test subject in the tub. PALs at all stimulus frequencies and all noise conditions at hearing threshold levels of the fish were determined with the acceleration sensor oriented in vertical direction (the direction of the sound presentation by the air speaker; up-down direction relative to the subjects). Control measurements in all three directions (up-down, left-right and rostral-caudal relative to the subjects) revealed that the contribution of other directions to the combined PAL was <1 dB (see also table 3 in [29]). We therefore focused on the vertical direction only. SPLs were calculated in dB re 1 µPa and PALs in dB re 1 µm/s^2^ (Table 1). These are the international units for sound pressure and particle acceleration according to ISO standards [35].

**Table 1:** Mean hearing thresholds of *E. maculatus* and *S. tinanti* at different frequencies and noise conditions.

***E. maculatus***					
Frequency (kHz)	0.2	0.5	1	2	3
**SPL** (dB re1 µPa)					
Baseline	77.2	71.3	75.8	110	114.5
WN 110 dB	86.2	79.2	87.3	111.7	114
WN 130 dB	106.3	97.8	101.7	124	129
**PAL** (dB re1 µm/s^2^)					
Baseline	33.6	39.4	37.7		
WN 110 dB	75.8	71.9	75.6		
WN 130 dB	75.8	71.9	75.6		
***S. tinanti***					
Frequency (kHz)	0.1	0.2	0.3	0.5	
**SPL** (dB re1 µPa)					
Baseline	99	92	96	110.8	
WN 110 dB	101	94	96.5	111.8	
WN 130 dB	104.8	108	110.8	116.5	
**PAL** (dB re1 µm/s^2^)					
Baseline	61.2	48.4	54.3	78.85	
WN 110 dB	63.3	52.8	54.8	80.15	
WN 130 dB	67.05	67.2	68.85	84.5	

SPL: note different frequency ranges for both species. No PAL-thresholds are given at 2 and 3 kHz. WN - white noise.

### Masking Noise Presentation and Noise Measurement

Audiograms were measured under normal laboratory conditions and in the presence of continuous white noise at two different levels. Masking noise was created by Cool Edit 2000, sent to a 30-band equalizer (Alesis MEQ 230) to obtain a flat noise spectrum underwater and fed to the second channel of a signal mixer (SM5 of TDT System 3) (for the equalizing effect see Fig. 1). The tone burst signals were fed to the first channel of the signal mixer. Both signals were then fed via the Alesis RA 300 amplifier to the dual-cone speaker (Tannoy System 600).

The SPLs of the masking noise were measured at the position of the fish using a sound level meter (Brüel & Kjaer 2238 Mediator) connected via a hydrophone power supply (Brüel & Kjaer 2804) to the hydrophone (Brüel & Kjaer 8101). We determined L-weighted (5 Hz to 20 kHz) equivalent continuous SPL (L_Leq_) averaged over 1 min measuring time. The Leq is a measure of the averaged energy in a varying sound level and commonly used to assess environmental noise. The system was calibrated using a Brüel & Kjaer 4229 calibrator. The L_Leq_s of the noise masker were 110 and 130 dB re 1 µPa. In addition, background noise levels in the experimental test tank (normal laboratory conditions) were measured. After SPL measurements, the background noise and the white masking noise were recorded via an external sound card (Cakewalk UA-25 EX) on a PC. Recording and analyzing were done using S_Tools-STX 3.7.8, an acoustics, speech, and signal processing application developed by the Acoustics Research Institute at the Austrian Academy of Sciences. Sound spectra of 1 min recordings were calculated by an FFT analysis using a filter bandwidth of 1 Hz. Absolute spectral values were calculated from the relative spectral values after calibrating the sound recording system (sound card, PC, software) using the calibrator (Brüel & Kjaer 4229).

Relative spectral PALs of the white noise were determined by recording the white noise via the p-a sensor in the vertical direction (see above). Absolute spectral PALs were calculated from the relative values using the sensor sensitivity and the calibration factor of the sound recording system.

### Statistical Analyses

As the assumption of normal distribution was met, parametric tests were applied. To determine if the presentation of masking noise resulted in significant threshold shifts between different noise conditions (lab noise, 110 dB and 130 dB white noise) repeated measures ANOVA was calculated at each frequency followed by LSD post hoc tests.

## Results

### Effects of Noise on Hearing Sensitivity in *Etroplus maculatus*

**Figure 2:**
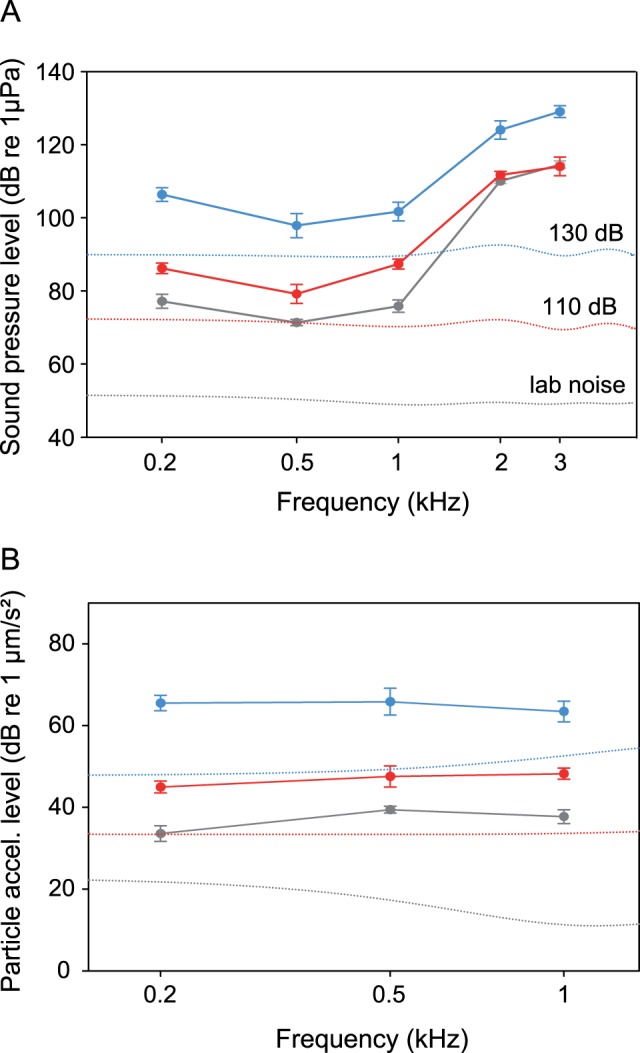
Mean (± S.E.) hearing thresholds of *Etroplus maculatus* obtained under quiet laboratory and different masking noise conditions (white noise of 110 and 130 dB). A) SPL and B) PAL audiograms. Dotted lines show cepstrum-smoothed spectra at different noise conditions.

**Figure 3:**
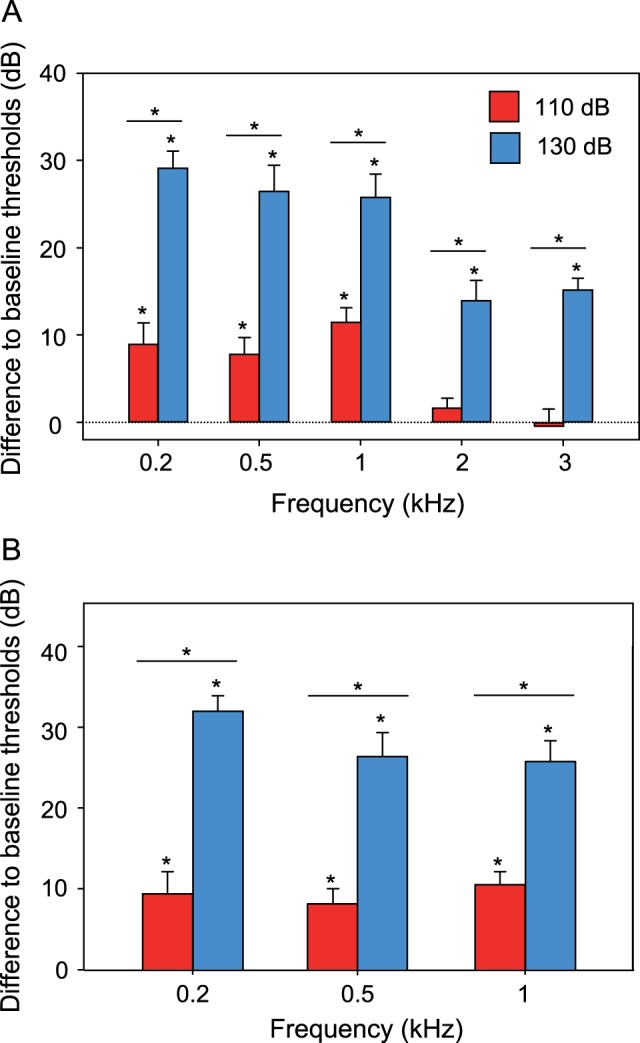
Mean (+ S.E.) difference between thresholds determined under white noise (110 dB and 130 dB) and under quiet laboratory conditions (baseline) of *E. maculatus*. Differences in A) SPL and B) PAL. Asterisks above bars indicate significant differences between baseline thresholds and masked conditions according to repeated measures ANOVA and LSD post hoc test (p<0.05). Asterisks above horizontal line indicate significant difference between masked conditions.

The baseline SPL-audiogram of *E. maculatus* revealed greatest hearing sensitivity between 0.2 and 1 kHz, with a steep decrease at higher frequencies (threshold at 500 Hz: 71.3 dB re 1 µPa) (Fig. 2A). At a masking noise level of 110 dB L_Leq_, the mean hearing sensitivity decreased significantly between 0.2 and 1 kHz by up to 12 dB, but not at the higher frequencies (Fig. 3A). At a noise level of 130 dB, significant sensitivity shifts up to 29.2 dB (at 200 Hz) were observed at all five frequencies investigated (Fig. 2A, 3A) (Table 1).

The baseline PAL-audiogram of *E. maculatus* showed highest sensitivity at 200 Hz (33. 6 dB re 1 µm/s^2^) and a significant decrease in hearing sensitivity at both masking noise levels at all frequencies measured (Fig. 2B, 3B). At the lower masking noise level, PAL-sensitivity shifted on average by 9.7 dB, and at the higher noise level on average by 28 dB (Table 1). Threshold shifts were significantly larger at 130 dB as compared to 110 dB at all frequencies tested (SPL and PAL).

### Effects of Noise on Hearing Sensitivity in *Steatocranus tinanti*

**Figure 4:**
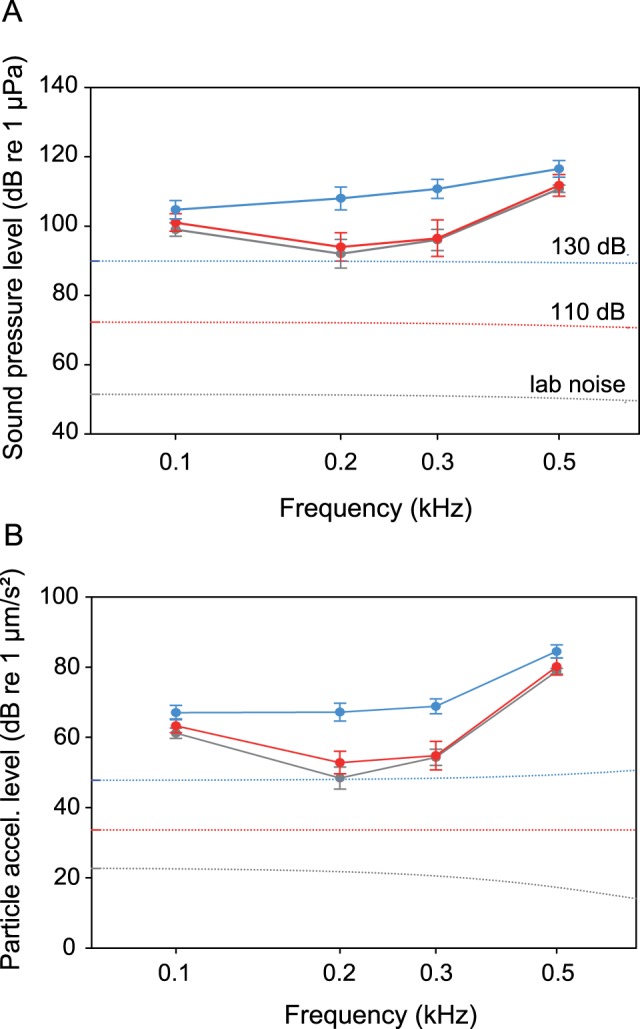
Mean (± S.E.) hearing thresholds of S*teatocranus tinanti* obtained under quiet laboratory and different masking noise conditions (white noise of 110 and 130 dB). A) SPL and B) PAL audiograms. Dotted lines show cepstrum-smoothed spectra at different noise conditions.

The cichlid *S. tinanti* had lower auditory sensitivities than *E. maculatus*, with the maximum sensitivity at 0.2 kHz (SPL: 92 dB re 1 µPa) (Fig. 4A). In contrast to *E. maculatus*, white noise of 110 dB did not affect the auditory sensitivity at any frequency. When animals were exposed to the 130 dB noise level, the sensitivity shifted significantly at 0.1, 0.2 and 0.3 kHz by maximally 16 dB (Fig. 4A, 5A) (Table 1).

**Figure 5:**
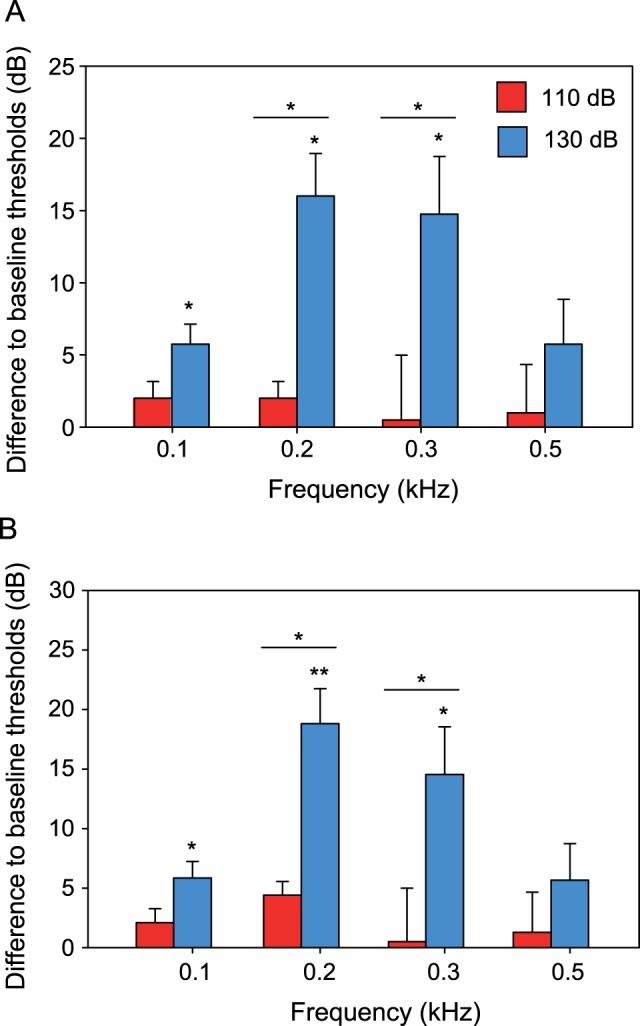
Mean (+ S.E.) difference between thresholds determined under white noise (110 dB and 130 dB) and under quiet laboratory conditions (baseline) of *S. tinanti*. Differences in A) SPL and B) PAL. For statistics see figure 3.

The baseline PAL-curve showed highest auditory sensitivity at 0.2 kHz (PAL: 48.4 dB re 1 µm/s^2^) (Fig. 4B). Again, no change in sensitivity was observed at the lower masking noise level (Fig. 5B). Exposure to the higher white noise level resulted in a decrease in sensitivity at three out of four frequencies. The maximum sensitivity shift occurred at 0.2 kHz, where this species showed its highest sensitivity (Fig. 5B) (Table 1). Threshold shifts were significantly larger at 130 dB as compared to 110 dB in 2 out of 4 frequencies (0.2 and 0.3 kHz, PAL and SPL).

## Discussion

The large diversity in hearing sensitivities even among closely related fish species (holocentrids and cichlids) raises the question of the functional significance of this diversity. Masking studies such as the present one help to elucidate this diversity, which was first recognized in the 1930s [36].

As discussed by Ladich [37], [38] it is unlikely that acoustic communication is the main selective force resulting in hearing enhancement because, in fishes, the ability to vocalize is independent of their hearing sensitivities. This notion is supported by vocalizing species such as gobies, toadfish and sculpins which lack accessory hearing structures, whereas non-vocal species like the majority of cypriniforms possess morphological specializations for hearing [37]–[39].

It is likely that eco-acoustical constraints, namely low background noise levels, resulted in the evolution of hearing enhancements, enabling fish species with such enhancements to detect low level sounds [12]. Enhanced hearing sensitivities may have been advantageous in detecting sounds from con- and heterospecifics for communication as well as orientation purposes, e.g. for detecting feeding conspecifics, prey or predators. This implies that species lacking morphological specializations for hearing live at higher ambient noise levels, whereas fishes possessing accessory hearing structures such as anterior swim bladder extensions should thrive in quiet habitats. In addition, Rogers and Cox [40] argued that high frequency hearing evolved in species living in shallow waters where the propagation of low frequencies is limited.

Rheophilic species such as the cichlid *S. tinanti* live in waters that are noisier than stagnant ones [8] and subsequently will not be able to utilize enhanced auditory sensitivities. In contrast, the cichlid *E. maculatus* inhabits more stagnant and thus more quiet waters and will be able to use its improved hearing abilities.

### Masking Effects within the Cichlid Family

The current study reveals that white noise presented at levels typically encountered in natural habitats affects hearing in different representatives of the cichlid family differently, and that this effect depends on absolute hearing sensitivities. In *E. maculatus*, hearing thresholds were significantly masked at their most sensitive frequencies (up to 1 kHz) at the lower masker level (SPL and PAL). Masking noise of 110 dB did not result in masking effects at 2 and 3 kHz because this species is approximately 30 dB less sensitive at these frequencies. A 20 dB increase in white noise (110 vs. 130 dB) increased the masked hearing thresholds linearly at the most sensitive frequencies. In contrast, *S. tinanti* was not affected by low levels of white noise at any frequency. At the higher noise level, hearing in *S. tinanti* was masked at the most sensitive frequencies (between 0.1 and 0.3 kHz; SPL and PAL). The loss in auditory sensitivity at 130 dB as compared to baseline levels is much smaller in *S. tinanti* than in *E. maculatus.*

A comparison of SPL and PAL threshold shifts reveals similar trends for both acoustic variables. This indicates that masking data based solely on SPL thresholds in prior studies on non-specialized species have a certain validity.

### Comparison between Cichlids and other Fish Taxa

In our intra-familial masking study, specialized and non-specialized species distinctly differed in the masking effect. Ramcharitar and Popper (2004) [17], however, came to a different conclusion when exposing two species of Western Atlantic sciaenids – the black drum *Pogonias chromis* and the Atlantic croaker *Micropogonias undulatus* – to two different levels of white noise (124 and 136 dB). The swim bladder of the black drum has no anterior projections, while that of the Atlantic croaker has extensions that approach the inner ears. The two species did not differ in absolute hearing sensitivities, indicating that the Atlantic croaker represents an intermediate form between fish with non-specialized and those with specialized swim bladders [41]. The black drum responded to tone bursts up to 0.8 kHz, whereas the Atlantic croaker could detect sound up to 1 kHz. In the presence of white noise of 124 dB, hearing thresholds shifted up to 10 dB in both species. Interestingly, although baseline thresholds and masked thresholds at the lower noise level were similar between 300 and 600 Hz in both species, the higher masker level only affected the black drum but not the Atlantic croaker. An increase in the white noise level of 12 dB resulted in an additional 10 dB hearing threshold shift in the black drum, whereas no such shift was observed in the Atlantic croaker. Those authors observed that the maximum frequency detectable decreased in the black drum to 700 Hz at 124 dB and to 600 Hz at a 136 dB noise level.

These observations in sciaenids contradict previous studies and the current data in cichlids. It is unexpected that two species exhibiting similar baseline thresholds are similarly affected by a certain noise level (124 dB) and differently affected by a 12 dB higher noise level. In the goldfish *Carassius aur*atus and the striped Raphael catfish *Platydoras armatulus*, which have similar baseline sensitivities (approximately 70 dB at 500 Hz) but belong to different orders within otophysines, a 20 dB increase in white noise level (110 dB vs. 130 dB) resulted in a threshold shift of approximately 20 dB at the most sensitive hearing range between 0.2 and 2 kHz [18]. In the cichlid *E. maculatus*, which is specialized for hearing similar to otophysines due to anterior swim bladder extensions, a similar shift in thresholds of approximately 20 dB was observed between 0.2 and 1 kHz. The Atlantic croaker possesses anterior swim bladder extensions that do not directly contact the inner ears [42] and differs morphologically from species specialized for sound pressure hearing such as otophysines and the cichlid *E. maculatus.* This may explain why it does not reveal linear threshold shifts similar to other hearing specialized fish. Ramcharitar and Popper (2004) [17] argued that the two sciaenids species differ in frequency selectivity because the Atlantic croaker was less susceptible to auditory threshold shifts, particularly at the higher levels of masking.

Our intra-familial results in cichlids are in good agreement with masking studies that compared species specialized for hearing, such as otophysines, and species not specialized, such as centrarchids and percids [18], [21]. In both prior studies, specialized fish revealed a much larger threshold shift in the presence of (white) noise than non-specialized species. In goldfish and the striped Raphael catfish, the hearing threshold increased by up to 22 dB at (500 Hz) at 110 dB white noise masker level and by up to 44 dB at 130 dB masker level [18]. In contrast, in the pumpkinseed sunfish *Lepomis gibbosus*, which had lower auditory sensitivities compared to both otophysines, with maximum sensitivity of 98 dB at 100 Hz, white noise of 110 dB did not affect its hearing sensitivity at any frequency. When sunfish were exposed to 130 dB noise level, the thresholds shifted up to 11 dB (500 Hz) compared to baseline thresholds.

Amoser and Ladich (2005) [21] recorded four ambient noise types in the field in eastern Austria (backwater: 91 dB RMS, lake: 93 dB, stream: 114 dB, river: 132 dB) and measured masking in the common carp *Cyprinus carpio* and the European perch *Perca fluviatilis.* The former is an otophysine and the latter is a non-specialized percid. Again, the same noise types resulted in quite different masking effects. In the carp, hearing sensitivity (highest sensitivity: ∼60 dB) declined at all types of habitat noise but particularly at the highest level by up to 49 dB. In contrast, the perch was less sensitive than the carp (baseline hearing thresholds 20 dB higher than that of the common carp). The presence of different masking noise types had slight (0–7 dB) or moderate effects on the hearing thresholds. Hearing thresholds were maximally elevated by 12 dB (200 Hz) in the case of the river noise.

### Biological Implications of Ambient Noise Masking in Fish

The above intra-familial and inter-familial comparative studies focused on the effects of noise on different hearing abilities. Several other studies analyzed the degree of masking under natural ambient noise conditions. The general observation was that masking effects (threshold shifts as compared to baseline thresholds) were small under quiet conditions such as in the absence of wind, rain or surf. Major threshold shifts were found under noisy conditions in fish possessing higher hearing abilities.

Chapman and Hawkins (1973) [20] and [19] measured hearing in the Atlantic cod *Gadus morhua* and other representatives of the family Gadidae in a Scottish Loch 15 m below the sea surface and 5 m above the sea bed. Unmasked thresholds were obtained only at calm sea conditions. More recent studies on non-related marine fish taxa such as batrachoidids, sciaenids, pomacentrids and gobiids revealed that the hearing sensitivities were only slightly masked. Vasconcelos et al. (2007) [23] showed that ambient noise from the Tagus River estuary in Portugal affected the auditory sensitivity only at low frequencies (50–100 Hz) in the Lusitanian toadfish *Halobatrachus didactylus* compared to quiet lab conditions. Codarin et al. (2009) [24] observed that the hearing sensitivity in the red-mouthed goby *Gobius cruentatus*, the brown meagre *Sciaena umbra* (family Sciaenidae) and the Mediterranean damselfish *Chromis chromis* (Pomacentridae) changed by less than 3 dB when exposed to the ambient noise recorded in their habitat, the Miramare Natural Marine Reserve in the Adriatic Sea. Observations in freshwater fishes revealed similar trends. Amoser and Ladich (2005) [21] showed that masking effects are low in quiet parts or periods of the common carp’s habitat (backwater, lake, no wind). This was supported by data in additional representatives of cyprinids. The topmouth minnow *Pseudorasbora parva*, a common Eurasian cyprinid, has best hearing sensitivities between 300 and 800 Hz (57 dB re 1 µPa) under quiet lab conditions. In the presence of ambient noise of its natural habitat (Lake Neusiedl), best thresholds shifted maximally 15 dB [22]. Gutscher et al. (2011) [25] showed that pond noise – a semi-artificial habitat – did not affect hearing in the goldfish. In contrast, pronounced masking effects were observed in the Atlantic cod [19]–[20] at high levels of wind, and in the common carp in noisy running waters [21].

Why did fish evolve enhanced hearing abilities when their hearing may be masked in their natural habitats? Accessory hearing structures and thus enhanced sensitivities may have evolved to enable fish in calm habitats to detect sound of different origin such as from conspecifics (feeding noise, territory advertising males, potential mates and opponents), heterospecifics (predators, prey) or abiotic sources (wind, surf, flowing water). Detecting and intercepting these sound sources at larger distances either permanently or during calm periods or in quiet regions of their distribution range may have been a major selective advantage over less specialized species. On the other hand, the presence of large swim bladders can be disadvantageous for bottom-dwelling fish due to buoyancy, particularly in running waters (cf. [43]). If these habitats were in addition noisy, then no counter-selective force will have acted on these bladders and they may have been subsequently reduced, e.g. in the cichlid *S. tinanti* or in the round goby *Neogobius melanostomus* [29], [44]. Such tendencies can be observed even in fish specialized for hearing such as loricariid and callichthyid catfishes, which belong to otophysines. Representatives of these bottom-living families possess tiny paired and encapsulated air bubbles located in the occipital region of the skull. This reduces their hearing abilities above 1 kHz [45].

In summary, the present study demonstrates that hearing sensitivities and subsequently masking can differ considerably within members of one bony fish family. This indicates that they live under different eco-acoustical conditions, i.e. different ambient noise regimes. Based on masking studies, we suggested that eco-acoustical constraints are probably the main selective forces shaping hearing sensitivities in fishes.
